# Kinetic Modelling of Infection Tracers [^18^F]FDG, [^68^Ga]Ga-Citrate, [^11^C]Methionine, and [^11^C]Donepezil in a Porcine Osteomyelitis Model

**DOI:** 10.1155/2017/9256858

**Published:** 2017-10-09

**Authors:** Lars Jødal, Svend B. Jensen, Ole L. Nielsen, Pia Afzelius, Per Borghammer, Aage K. O. Alstrup, Søren B. Hansen

**Affiliations:** ^1^Department of Veterinary and Animal Sciences, University of Copenhagen, Copenhagen, Denmark; ^2^Department of Nuclear Medicine and PET Centre, Aarhus University Hospital, Aarhus, Denmark; ^3^Department of Nuclear Medicine, Aalborg University Hospital, Aalborg, Denmark; ^4^Department of Chemistry and Biosciences, Aalborg University, Aalborg, Denmark; ^5^Department of Diagnostic Imaging, North Zealand Hospital, Copenhagen University Hospital, Hillerød, Denmark

## Abstract

**Introduction:**

Positron emission tomography (PET) is increasingly applied for infection imaging using [^18^F]FDG as tracer, but uptake is unspecific. The present study compares the kinetics of [^18^F]FDG and three other PET tracers with relevance for infection imaging.

**Methods:**

A juvenile porcine osteomyelitis model was used. Eleven pigs underwent PET/CT with 60-minute dynamic PET imaging of [^18^F]FDG, [^68^Ga]Ga-citrate, [^11^C]methionine, and/or [^11^C]donepezil, along with blood sampling. For infectious lesions, kinetic modelling with one- and two-tissue-compartment models was conducted for each tracer.

**Results:**

Irreversible uptake was found for [^18^F]FDG and [^68^Ga]Ga-citrate; reversible uptake was found for [^11^C]methionine (two-tissue model) and [^11^C]donepezil (one-tissue model). The uptake rate for [^68^Ga]Ga-citrate was slow and diffusion-limited. For the other tracers, the uptake rate was primarily determined by perfusion (flow-limited uptake). Net uptake rate for [^18^F]FDG and distribution volume for [^11^C]methionine were significantly higher for infectious lesions than for correspondingly noninfected tissue. For [^11^C]donepezil in pigs, labelled metabolite products appeared to be important for the analysis.

**Conclusions:**

The kinetics of the four studied tracers in infection was characterized. For clinical applications, [^18^F]FDG remains the first-choice PET tracer. [^11^C]methionine may have a potential for detecting soft tissue infections. [^68^Ga]Ga-citrate and [^11^C]donepezil were not found useful for imaging of osteomyelitis.

## 1. Introduction

Positron emission tomography (PET) allows imaging of molecular uptake but is dependent on the availability of tracers with uptake related to the investigated disease. For infection imaging, [^18^F]FDG is useful [1], but a drawback is the nonspecific uptake of [^18^F]FDG in metabolically active tissues (brain, muscles, etc.) [2].

We previously reported on static PET imaging in a porcine osteomyelitis model with a series of tracers that have been proposed for infection imaging [3–5]. In those reports, we concluded that the performance of [^18^F]FDG for locating infection was superior to the other tracers studied. However, our static imaging shows only the uptake at a given (typically late) time interval.

In the present paper, we elaborate on those studies [3–5] by including data from* dynamic* PET imaging of the same animals (based on the same tracer injections), using kinetic analysis to compare [^18^F]FDG with [^68^Ga]Ga-citrate, [^11^C]methionine (l-[^11^C-*methyl*]methionine), and [^11^C]donepezil ([5-^11^C-*methoxy*]donepezil) as infection tracers. Dynamic imaging allows for a more detailed study of the uptake and release of tracers, which may contribute to a better understanding of the underlying physiology and also help in determining the optimal time for static imaging. Blood perfusion results from dynamic imaging of [^15^O]water in the same animals have already been reported [6].

Gamma-camera imaging (scintigraphy or SPECT) of infection is sometimes performed with [^67^Ga]Ga-citrate. However, image quality is suboptimal and the radiation dose from ^67^Ga is high. The positron-emitter ^68^Ga allows PET imaging, which has a better spatial resolution and a higher detection efficiency than standard gamma-camera imaging. Furthermore, the shorter half-life of ^68^Ga (67.7 min versus 78 hours for ^67^Ga) reduces the radiation dose. In the body, the Ga^3+^ ion acts as an analogue of iron (Fe^3+^), associating with transferrin; for more details, see [7]. Preliminary studies on [^68^Ga]Ga-citrate in rats [8] and patients [9, 10] reported promising results.

Methionine is a naturally occurring essential amino acid. It can be labelled with the positron-emitter ^11^C to obtain the chemically identical PET tracer [^11^C]methionine. Methionine (natural or labelled) is transported into cells via the L-type amino acid transporter 1. It is crucial for the formation of proteins and is involved in the synthesis of phospholipids. The accumulation of methionine therefore reflects amino acid transport and protein synthesis. During cell replication, the demand for essential amino acids increases, as do protein and phospholipid synthesis. Although primarily used to detect malignant tumours [11], methionine is known from brain studies to accumulate in inflammatory tissue [12, 13]. Furthermore, a high uptake of [^11^C]methionine has been observed after acute myocardial infarction, indicating that this tracer may be a good marker of inflammatory reactions during the phase of tissue repair [14].

Donepezil is a reversible inhibitor of acetylcholinesterase (AChE), the break-down enzyme of acetylcholine (ACh) [15]. ACh and AChE are primarily known as constituents in cholinergic neuronal signalling pathways, but immune cells have been shown to use ACh as a paracrine signalling molecule [16], and AChE and cholinergic receptors are upregulated in immune cells when exposed to phytohaemagglutinin [17]. It was recently shown that the AChE ligand [^11^C]donepezil accumulates at sites of bacterial infection, suggesting that cholinergic PET imaging may have potential in the detection of inflammation and infections [18].

Therefore, we investigated the uptake processes of these PET tracers in order to determine their clinical potential in infection imaging in relation to both osteomyelitis and soft tissue infection. We hypothesized that (1) categorization of tracers into those having either reversible or irreversible uptake in infectious lesions is possible and (2) perfusion effects on tracer accumulation can be estimated. Finally, we wanted to estimate the optimal time points for static imaging of the tracers.

## 2. Materials and Methods

### 2.1. Porcine Osteomyelitis Protocol

The protocol for inducing osteomyelitis in domestic pigs has been described in detail elsewhere [3, 19–21]. Briefly, osteomyelitis was induced in the right hind limb of juvenile Danish Landrace × Yorkshire female pigs by intra-arterial injection of* S. aureus* (porcine strain S54F9). The injection was performed into the right femoral artery, allowing for selective infection of this hind limb while leaving the contralateral left hind limb as a noninfected control. Osteomyelitis was allowed to develop for one week, after which the pig was scanned and then euthanized. If a pig reached predefined humane endpoints, it was then euthanized (and not scanned). A refinement of the model, combining reduced body weight of the pigs (from 40 kg to 20 kg) with administration of penicillin at the onset of the first clinical signs of disease, proved effective in reducing systemic infection [21].

We attempted to prolong the infection period from one to two weeks, for the purpose of developing more chronic lesions, and managed to scan one pig two weeks after inoculation (pig number 5 of the 11 scanned pigs described below). However, 4 out of 5 pigs planned for scanning with the prolonged protocol had to be prematurely euthanized due to humane endpoints, so we returned to the subacute one-week pig protocol.

The animal protocol was approved by the Danish Animal Experimental Board, journal number 2012-15-2934-00123, and all procedures followed the European Directive 2010/63/EU on the protection of animals used for scientific purposes.

### 2.2. Animals and Lesions

Eleven juvenile female domestic pigs were scanned. Pigs number 1–4 had body weights of 39–42 kg (initial model) and pigs number 5–11 had body weights of 19–23 kg (refined model). Blood perfusion in the lesions in these pigs has been described previously [6], including 17 osteomyelitic (OM) lesions and 8 soft tissue (ST) lesions, with volume of interest (VOI) drawing primarily based on computed tomography (CT) scans. Other OM lesions were also found in pedal bones but were too small for robust volume of interest (VOI) drawing.

In the present paper, the kinetics of [^18^F]FDG, [^68^Ga]Ga-citrate, [^11^C]methionine, and [^11^C]donepezil were studied and modelled in the same 17 OM lesions and 8 ST lesions, with VOIs redrawn on the CTs from the dynamic PET/CT scans of these tracers. Not all tracers were used in all pigs, and the limitations set by the PET scanner field of view (FOV, further described below) resulted in not all lesions being dynamically scanned in all pigs. An overview is given in Table 1.

We have previously reported some data on the characterization of the lesions evolving from* S. aureus* inoculation in these pigs, including the methods used for bacteriological and immunohistochemical (IHC) identification of bacteria [3–5]. Generally, the lesions observed in the pigs were considered to be caused by infection associated with the inoculated* S. aureus* if the bacterial culture and/or* S. aureus* specific IHC staining confirmed the presence of* S. aureus* in one or several of the lesions within each individual pig (results not shown).

### 2.3. Dynamic PET Scans

PET scans were carried out at the Department of Nuclear Medicine & PET Centre (Aarhus University Hospital), and at Department of Nuclear Medicine (Aalborg University Hospital), with the pigs transported from one hospital to the other. The PET scans at Aarhus included the ^11^C-labelled tracers in pigs number 1–10 and [^18^F]FDG in pig number 11. The PET scans at Aalborg included [^68^Ga]Ga-citrate in pigs number 1–5 and [^18^F]FDG in pigs number 1–10.

At Aarhus, the PET data were acquired on a Biograph TruePoint 64 PET/CT scanner (Siemens, Erlangen, Germany). The scan field covered 21 cm in the axial direction and was positioned over the pelvic region and the hind limbs. The images were reconstructed with an OSEM algorithm with resolution recovery (TrueX, Siemens). The reconstruction parameters were 6 iterations, 21 subsets, 336 × 336 matrix in 109 slices, voxel size 2 × 2 × 2 mm^3^, and a 2 mm Gaussian filter. The spatial resolution of the reconstructed images was approximately 4 mm.

At Aalborg, the PET data were acquired on a GE VCT Discovery 64 PET/CT scanner (GE Healthcare, USA). The scan field covered 15 cm in the axial direction and was positioned over the pelvic region and the hind limbs. The images were reconstructed with an OSEM algorithm without resolution recovery (3D Vue Point, GE). The reconstruction parameters were 2 iterations, 28 subsets, 128 × 128 matrix in 47 slices, voxel size 5.5 × 5.5 × 3.3 mm^3^, and a 6 mm Gaussian filter.

On both scanners, image reconstruction included decay-correction to the start of scanning and attenuation-correction based on CT scanning.

For all tracers, the pigs were dynamically PET scanned for 60 minutes in 23 frames: 8 × 15 s, 4 × 30 s, 2 × 60 s, 2 × 120 s, 4 × 300 s, and 3 × 600 s. For [^68^Ga]Ga-citrate, the animals were scanned for an additional 6 × 600 s (i.e., 120 minutes total scan time in 29 frames). After each of the dynamic scans, the pigs were statically scanned; the static PET/CT scans have been described in previous papers [3–5] and will not be further discussed here.

**Figure 1:**
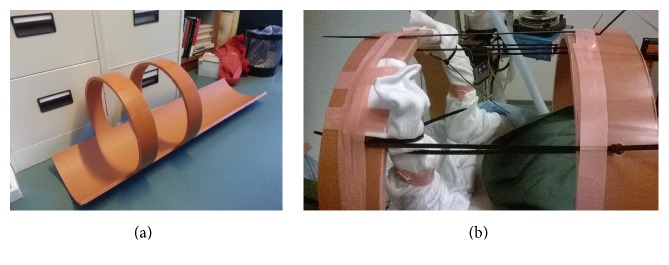
Two fixation devices for the pigs were produced from large plastic tubes, with a distance between the arches of either 15 or 21 cm, corresponding to the scanner FOVs. Assuming correct positioning of the device in the scanner, the arches made it visually clear where the FOV of the PET scan was located, thereby facilitating optimal positioning in the scanner. The fixation device also facilitated symmetric limb positioning and the avoidance of limb movement during the long scan sessions. (a) The 21 cm FOV fixation device. (b) Pig number 6 in the device.

The pigs were scanned in dorsal recumbency (supine position). The hind limbs were positioned for the entire hind limbs and pelvis to be within the axial field of view (FOV) of the scanner. This was, however, not always possible; in particular, the 15 cm FOV at the Aalborg scanner (versus 21 cm FOV at the Aarhus scanner) was a limitation. To optimize the fixation position to the scanner FOV, custom-made fixation devices were used for pigs number 6–11 (Figure 1).

### 2.4. Blood Samples

Blood samples were drawn from the carotid artery at predetermined time points as listed below. The samples were manually drawn, and small variations occurred; the actual time of each sample was recorded. The samples were centrifuged to obtain plasma samples, which were counted in calibrated gamma-counters.

At Aarhus, plasma samples were counted in a Packard Cobra gamma counter. An energy window from 400 to 1400 keV was used. No signs of interference between consecutively administered radionuclides were seen in the plasma curves.

At Aalborg, plasma samples and full-blood samples were counted in a Wizard 2480 gamma counter (PerkinElmer, Turku, Finland). To avoid interference from remnants of ^111^In from ^111^In-leukocytes (used in the same animals [3]), an energy window from 450 to 1200 keV was used [22].

In the 40 kg pigs (pigs number 1–4), 37 blood samples were drawn per tracer: every 5 seconds for 1 minute (12 samples), at 70, 80, 90, 100, 120, 140, 160, 180, 210, 240, 270, and 300 seconds (12 samples), and at 6, 7, 8, 9, 10, 15, 20, 25, 30, 35, 40, 50, and 60 minutes (13 samples). For [^68^Ga]Ga-citrate, blood samples were also drawn at 75, 90, 105, and 120 minutes (four samples), for a total of 41 blood samples.

In the 20 kg pigs (pigs number 5–11), the number of blood samples was reduced to 26 samples per tracer: every 5 seconds for 50 seconds (10 samples), at 60, 80, 100, 120, 150, 180, 240, and 300 seconds (8 samples), and at 6, 8, 10, 15, 20, 30, 40, and 55 minutes (8 samples).

For [^11^C]methionine and [^11^C]donepezil, additional blood samples were drawn for metabolite analysis: at 2, 5, 10, 15, 25, and 40 minutes in the 40 kg pigs, and at 2, 5, 10, 20, 30, 40, and 55 minutes in the 20 kg pigs.

### 2.5. Input Function

The blood plasma rather than full blood was considered to be the reference fluid for tracer delivery (corresponding to equilibration between blood cells and plasma being slow compared to single-passage time of blood in the tissue). Accordingly, all input functions were based on plasma samples.

For both [^18^F]FDG and [^68^Ga]Ga-citrate, the decay-corrected plasma sample data were used as the input function. No metabolite correction was performed for these two tracers because metabolite products are not expected to be found in the blood. [^18^F]FDG is phosphorylated within the cells, but the resulting (radioactive) metabolite is trapped within the cells [23]. Regarding [^68^Ga]Ga-citrate, the Ga-citrate complex quickly dissociates into Ga^3+^ and citrate^3-^ within the blood, but the gallium ion attaches to transferrin (making [^68^Ga]Ga-transferrin the actual tracer). Therefore, free gallium is not found in the blood [24].

For [^11^C]methionine and [^11^C]donepezil, metabolite correction was performed. A fractionated HPLC analysis was used to separate the metabolites from the parent tracer, and counting was used to determine the fraction of activity representing the parent tracer. Based on the obtained data points, Hill-type fraction curves were fitted:

(1) $$\begin{array}{c} f\left(\;t\;\right)=1-\frac{\left(1-a\right)t^{b}}{c+t^{b}}, \end{array}$$

where *t* is the sampling time (seconds postinjection). The function starts at *f*(0) = 1 (thus assuming no metabolism before injection) and has an asymptotic value *f*(*∞*) = *a*. The parameters *a*, *b*, and *c* were fitted for both tracers in the individual pigs.

In the following text, the* uncorrected input function* will denote the activity concentration (decay-corrected Bq/mL) from plasma samples and the* metabolite-corrected input function* will denote *f*(*t*) times the uncorrected input function.

### 2.6. Determination of the Delay-Correction

The measured input function can be biased by delay and dispersion effects due to the differences in distance between the blood sampling site (carotid artery) and the infection sites in the hind limbs. Such a delay can be determined by applying a series of possible delay-correction values, fitting the data with each value, and selecting the delay-correction resulting in the best fit [25].

**Figure 2:**
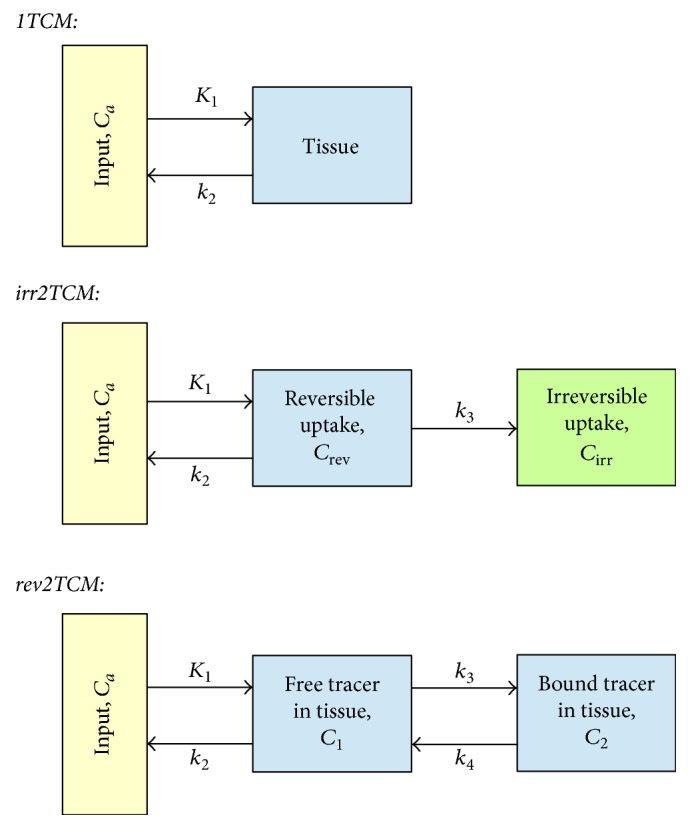
Kinetic models. From top to bottom: 1-tissue compartment model (1TCM), 2-tissue compartment model with irreversible uptake (irr2TCM), and 2TCM with reversible uptake (rev2TCM). The rate constants were fitted as *K*_1_ (unit mL/min/cm^3^ or mL/min/100 cm^3^), the ratio *K*_1_/*k*_2_ (unit mL/cm^3^), *k*_3_ (unit min^−1^), and the ratio *k*_3_/*k*_4_ (no unit). Also the blood fraction *V*_*b*_ was fitted.

We applied this procedure for each tracer in each animal, using input function offsets from −60 s to +60 s in 1-second steps. This was done using the uncorrected plasma data for the input function, and fitting a reversible two-tissue compartment model (rev2TCM in Figure 2; the models are further discussed below) to the first 300 s of the full field-of-view data. Using full FOV data ensures that the statistical noise is low. Restricting this fit to the early data has several advantages. First, this focuses on the part of the study where the input function changes fast and the delay therefore is important. Second, this means that physiological differences in uptake (e.g., bladder versus nonbladder, infection versus noninfection) will only have had little time to manifest; thus the full FOV data will be dominated by the bolus passage in this anatomical part of the animal, rather than by a mix of physiologies. Third, the possible metabolism effects will not yet dominate the input function, for which reason the uncorrected input function can be considered representative, even if the tracer over time is metabolized.

### 2.7. Kinetic Models

Relatively little literature exists on tracer kinetics in infections for the tracers investigated in this work. Rather than imposing a specific model on these tracers, we examined the applicability of three different models for each tracer (Figure 2).

Physiologically, the 1TCM corresponds to the tracer entering and leaving the tissue with no binding or other specific uptake. The irr2TCM describes uptake and irreversible trapping of the tracer or metabolites (e.g., phosphorylated [^18^F]FDG). The rev2TCM corresponds to uptake in the tissue followed by reversible binding (or reversible metabolism) of the tracer.

In all of the models, the *K*_1_ rate constant describes the first-pass uptake of tracer, equal to the product of perfusion and the first-pass extraction fraction. We report *K*_1_ in units of mL/min/100 cm^3^, that is, mL uptake per minute per 100 cm^3^ of tissue. The other rate constants (*k*_2_, *k*_3_, and *k*_4_, all with unit min^−1^) describe how rapid the concentration in a compartment changes due to a given process (excretion, binding, and metabolism in the tissue). The blood fraction *V*_*b*_ is the fraction of measured PET signal that originates from the blood in the vascular bed.

For models with irreversible uptake, the net uptake rate *K*_*i*_ (same unit as *K*_1_) represents the effective irreversible uptake from the input. Whereas *K*_1_ represents the rate of immediate (first-pass) uptake of tracer, *K*_*i*_ can similarly be interpreted as the rate of long-term uptake. For the irr2TCM, the theoretical net uptake rate is

(2) $$\begin{array}{c} K_{i}=\frac{K_{1}\cdot k_{3}}{k_{2}+k_{3}}\;\left(\text{for }irr2TCM\right). \end{array}$$

The net uptake rate may also be determined as the slope of a Patlak plot [26, 27].

For models with reversible uptake, the distribution volume (DV, unit mL/cm^3^) is the ratio of the tissue concentration to the “input” concentration, once a steady-state has been reached, that is, the volume of “input” needed to account for the activity in 1 cm^3^ of tissue [28]. Although not being an uptake rate, DV may be used as an indicator of the degree of long-term uptake. For the 1TCM and rev2TCM, the theoretical distribution volumes are

(3) DV=K1k2for  1TCM

(4) DV=K1k2·1+k3k4for  rev2TCM.

The distribution volume of reversible uptake may also be determined as the slope of a Logan plot [29, 30].

### 2.8. Weighting of the PET Data in Modelling

Theoretically, least-squares fitting is optimal with weights proportional to 1/*σ*^2^ where *σ*^2^ is the variance of the noise. With *N* counts (Poisson distributed) during a frame length *L*, the count rate *R* = *N*/*L* has variance:

(5) $$\begin{array}{c} \sigma^{2}\left(\;R\;\right)=\frac{N}{L^{2}}=\frac{R}{L}. \end{array}$$

After decay-correction:

(6) Rdc=dcf×Rσ2Rdc=dcf2×RL=dcf×RdcL,

where dcf is the decay-correction factor, calculated from the radionuclide half-life and frame time interval. For weighting purposes, it can be well approximated by using the mid-time of the frame:

(7) $$\begin{array}{c} dcf=2^{t/T_{1/2}}=\exp\left(\;\lambda t\;\right). \end{array}$$

Seemingly, optimal weighting should be 1/*σ*^2^ = *L*/*R* for non-decay-corrected data and 1/*σ*^2^ = *L*/(dcf × *R*_dc_) for decay-corrected data.

However, the count rate (or the activity concentration) is known only from a measurement that includes noise. In a simulation study, Thiele and Buchert [31] found that noise in the weighting factors can severely degrade parameter estimation and should therefore be avoided. Consistent with the weighting that gave the best results in that study, we used the following noise-free weighting factors for the decay-corrected PET data:

(8) $$\begin{array}{c} w=\frac{L}{dcf}=L\times \exp\left(\;-\lambda t\;\right). \end{array}$$

These weights correctly include the effects of decay and the large differences in frame length (from 15 to 600 seconds). The weights are noise-free, at the cost of ignoring the statistical effects from variation in tracer concentration due to kinetics.

This approximation (weights not fully reflecting 1/*σ*^2^) is unlikely to be a problem. A simulation study by Yaqub et al. [32] found that kinetic modelling of PET data was reasonably robust against some misrepresentation of the variance in (noise-free) weighting, with only severe misrepresentation being a problem.

### 2.9. Modelling

For each tracer in each model (1TCM, irr2TCM, and rev2TCM), the fitted parameters were determined using least-squares fitting with the weighting described above. Additionally, Patlak plots and Logan plots were computed, based on the data from 10 minutes postinjection (p.i.) and onwards.

For [^11^C]methionine and [^11^C]donepezil, this procedure was performed twice: using an uncorrected input function and using a metabolite-corrected input function. For these two tracers, modelling was restricted to data from the first 40 minutes (out of 60 minutes), as metabolite data were in many cases incomplete for later frames.

Modelling was performed using software acquired from the Turku PET Centre website [33]. The parameter *k*_2_ was fitted as the ratio *K*_1_/*k*_2_ (corresponding to the distribution volume of the first compartment). In the rev2TCM, the parameter *k*_4_ was fitted as the ratio *k*_3_/*k*_4_ (corresponding to the binding potential BP if the first and second compartments are considered to represent unspecific and specific uptake, resp.).

### 2.10. Evaluation

In addition to visual inspection of the fits, the three models were compared with the corrected Akaike Information Criterion (AIC_c_), which rewards a good fit but punishes the use of a model with many fitting parameters. For a given data set, AIC_c_ favours the model resulting in the lowest AIC_c_ value [34, 35].

For the determination of uptake as reversible or irreversible, the Patlak plot also was considered. If the uptake is reversible (i.e., not irreversible), the Patlak plot will eventually approach a constant value. Therefore, the linearity of the Patlak plot with a nonzero slope can be used as a test for irreversible uptake.

All these results are based on plasma input functions. To compare with perfusion, previously published results for blood perfusion in the same animals [6] were transformed into plasma perfusion by the formula

(9) plasma  perfusion=1−haematocrit×blood  perfusion,

with haematocrit being measured at a time roughly corresponding to the time of the [^15^O]water PET.

## 3. Results

### 3.1. Metabolite Correction

**Figure 3:**
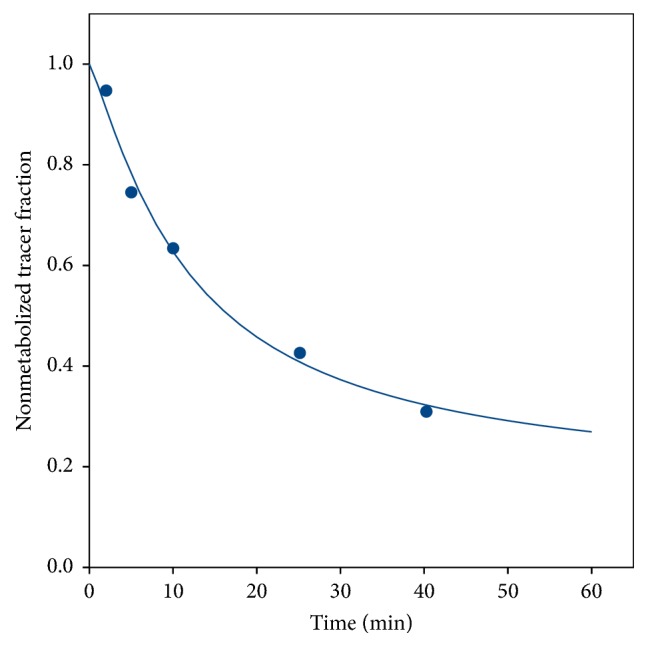
Representative data and curve fit (see (1)) for the fraction of nonmetabolized [^11^C]methionine (from pig number 1).

**Figure 4:**
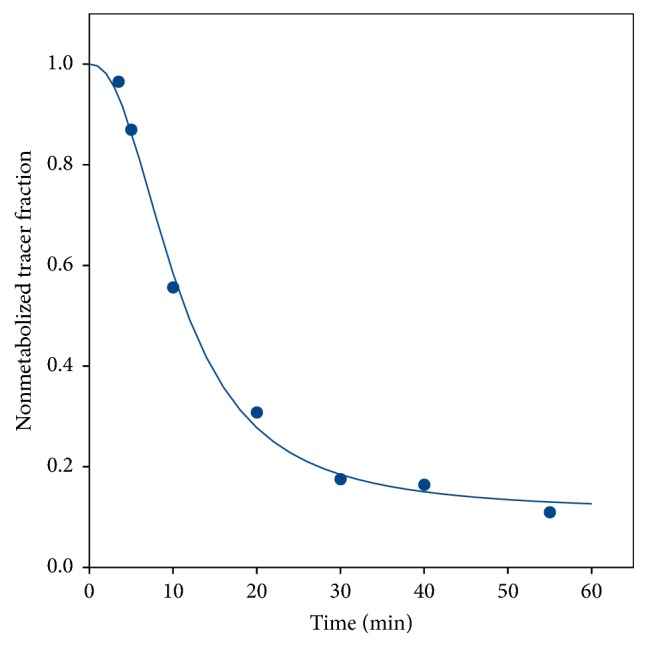
Representative data and curve fit (see (1)) for the fraction of nonmetabolized [^11^C]donepezil (from pig number 10).

The fraction curves indicated considerable metabolism of both [^11^C]methionine (Figure 3) and [^11^C]donepezil (Figure 4).

**Figure 5:**
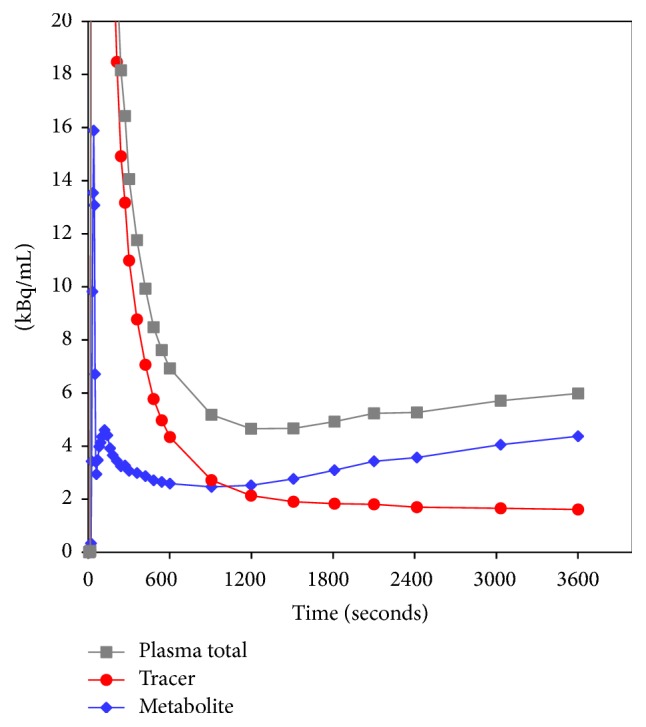
Representative sample plasma activity curves for [^11^C]methionine (decay-corrected, from pig number 1). The scale of the vertical axis has been chosen to emphasize the curve tails, at the cost of truncating the bolus peak (maximum value ~600 kBq/mL). Uncorrected input function is the total plasma activity concentration (grey curve). Metabolite-corrected input function (red curve) originates as the product of the total plasma activity curve and the fraction curve (Figure 3). Also shown is the metabolite activity concentration (blue curve), that is, the difference between the other two curves. Similar curves were seen for [^11^C]donepezil (not shown).

For both [^11^C]methionine and [^11^C]donepezil, the “uncorrected” (i.e., not corrected for metabolism, but corrected for physical decay) plasma curves showed an unexpected tendency of slightly rising values after typically 20 minutes. As an example, see the data for [^11^C]methionine in Figure 5 (the data for [^11^C]donepezil were similar). Possible reasons like unresolved background counts, either from other tracers in the multitracer study or from the surroundings, were investigated, but no sign of any such problems was found; for example, blood samples taken before the arrival of the bolus injection were far below the level that would cause this background. Therefore, we conclude that the curves correctly represent the activity concentration in the plasma. After metabolite correction, the curves decreased as expected (Figure 5).

### 3.2. Delay-Correction of Input Function

Each input function (*n* = 26) was individually delay-corrected (but with a common delay for all lesions in the same scan). The mean ± SD of the corrections was −4.5 ± 4.1 seconds. A negative correction corresponds to the tracer arriving earlier to the scanned tissue (PET data) than to the site of blood sampling (plasma input data). All individual corrections were numerically smaller than the initial PET frame length (15 seconds).

### 3.3. Modelling

The overall results for the different models are summarized in Table 2, with elaborating comments given here.

#### 3.3.1. [^18^F]FDG

**Figure 6:**
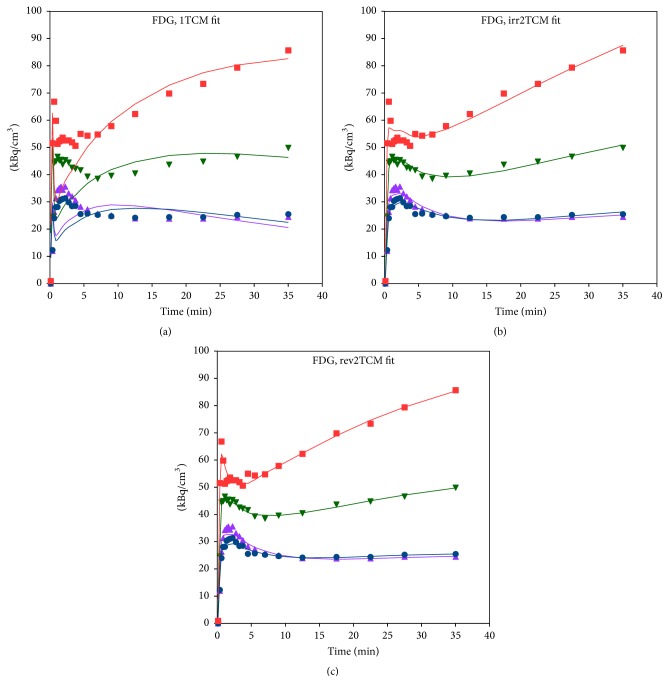
Model fits for [^18^F]FDG in pig number 6, using 1TCM (a), irr2TCM (b), and rev2TCM (c) models. Red and blue: OM lesion in the distal femur and the corresponding noninfected bone. Green and mauve: OM lesion in the proximal tibia and the corresponding noninfected tissue. In this case, modelling was restricted to 0–40 minutes because movement of the right limb occurred in the following frame.

Despite the lowest AIC_c_ values having been found for rev2TCM, the irr2TCM appeared visually to give a reasonable fit for the investigated time range. Example fits are shown in Figure 6. The Patlak plots (not shown) were linear with nonzero slopes, indicating the presence of irreversible uptake. Also, the majority of fitted ratios *k*_3_/*k*_4_ were above 2 (median value ~4 for all VOIs, ~5 if restricted to infected side); that is, overall *k*_4_ was considerably lower than *k*_3_. For these reasons and because irr2TCM has fewer parameters than rev2TCM, we pragmatically chose to base the further analysis of [^18^F]FDG uptake on the* irr2TCM*. The values of *K*_1_ were overall very similar for irr2TCM and rev2TCM.

#### 3.3.2. [^68^Ga]Ga-Citrate

**Figure 7:**
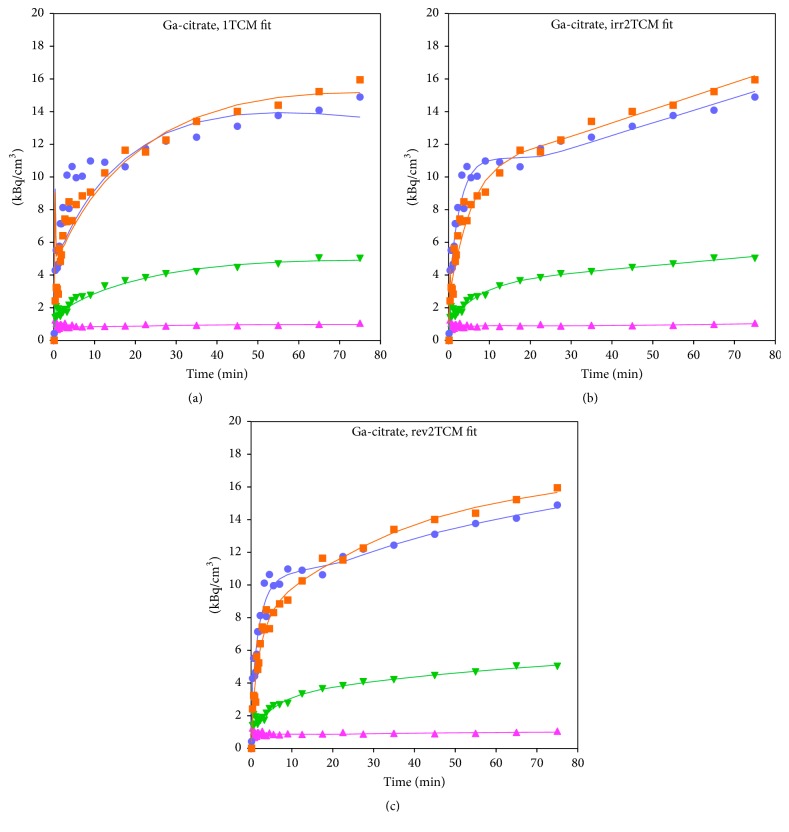
Model fits for [^68^Ga]Ga-citrate in pig no. 1, using 1TCM (a), irr2TCM (b), and rev2TCM (c) models. Orange and light blue: OM lesion in the proximal femur and the corresponding noninfected bone. Light green and cyan: ST lesion near the metatarsal bone and the corresponding noninfected tissue.

Overall, the AIC_c_ values indicated nearly equal quality of fits for irr2TCM and rev2TCM. Example fits are shown in Figure 7. The Patlak plots were linear with nonzero slopes. Further analysis will assume the simplest model:* irr2TCM*.

For both [^18^F]FDG and [^68^Ga]Ga-citrate, good correspondence was observed between the slope of the Patlak plot and *K*_*i*_ calculated from the irr2TCM parameters.

#### 3.3.3. [^11^C]Methionine

**Figure 8:**
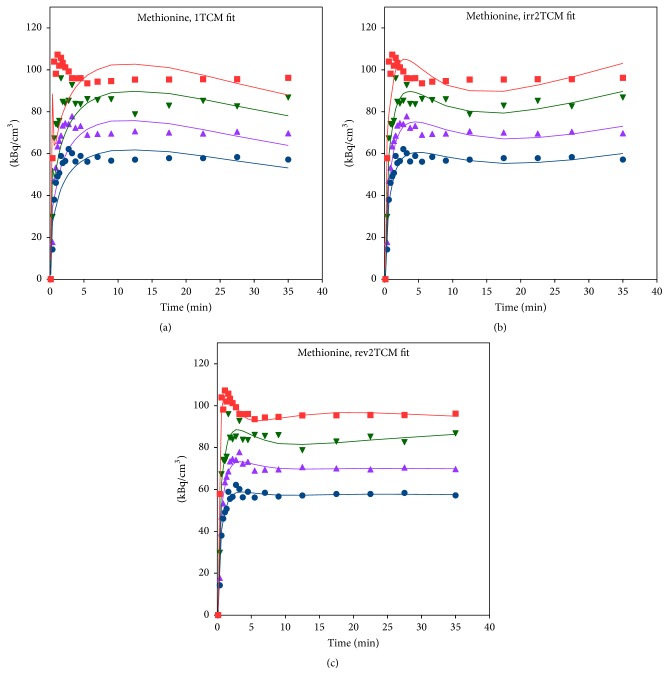
Model fits for [^11^C]methionine in pig number 6 (same animal as in Figure 6), using uncorrected plasma data as the input function. Legend as in Figure 6. Fits with a metabolite-corrected input function (not shown) were visually very similar to the shown fits, although with a reduced upward trend in the irreversible model (b).

The AIC_c_ values sometimes favoured rev2TCM and sometimes favoured irr2TCM, but, from mean the and median values, rev2TCM was favoured. Visually, the irr2TCM fit showed a problematic upward trend in the late part of the fits (Figure 8). Despite the extent of metabolism during the study (Figure 3), only a small difference was observed between using the uncorrected or the metabolite-corrected input function with the rev2TCM: the AIC_c_ values were comparable, the fits were visually very similar, and the fitted rate parameters *K*_1_, *K*_1_/*k*_2_, and *k*_3_ were very similar. Only the *k*_3_/*k*_4_ values differed markedly, being lower for the corrected than the uncorrected input function. However, the *k*_3_/*k*_4_ ratio is important for the distribution volume (see (4)), and the further analysis of the [^11^C]methionine data will assume the* rev2TCM model with a metabolite-corrected input function*.

#### 3.3.4. [^11^C]Donepezil

**Figure 9:**
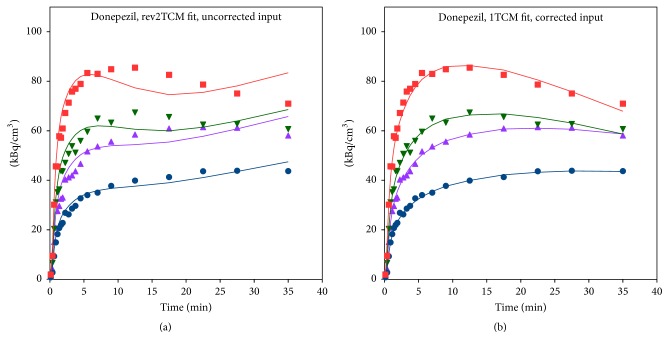
Model fits for [^11^C]donepezil in pig number 6 (same animal as in Figures 6 and 8). Legend as in Figure 6. (a) Using the* uncorrected* input function, even the model with the most parameters (rev2TCM) gives a poor fit. (b) Using the* metabolite-corrected* input function, the model with the least parameters (1TCM) gives a good fit.

Both the plots and the AIC_c_ values unequivocally favoured fits with a metabolite-corrected input function (Figure 9). Within these, the AIC_c_ values variably favoured each of the three models but with no obvious pattern (e.g., not distinguishing infected versus noninfected tissue, bone versus soft tissue, or high versus low *K*_1_). Visually, however, the 1TCM (with metabolite-corrected input) fits well in all cases. A typical fit for the 1TCM is seen in Figure 9(b). Further analysis of [^11^C]donepezil will be based on the* 1TCM with a metabolite-corrected input function*.

### 3.4. Perfusion and First-Pass Uptake Rate (*K*_1_)

The *K*_1_ parameter represents the product of perfusion and the extraction fraction for the tracer. Thus, plotting *K*_1_ as a function of perfusion gives a measure of extraction; if the extraction fraction is independent of perfusion, the plot will show proportionality between *K*_1_ and perfusion. The blood perfusion of these lesions in these animals has been described previously, based on [^15^O]water PET scans [6]. These data were transformed into plasma perfusion according to (9).

**Figure 10:**
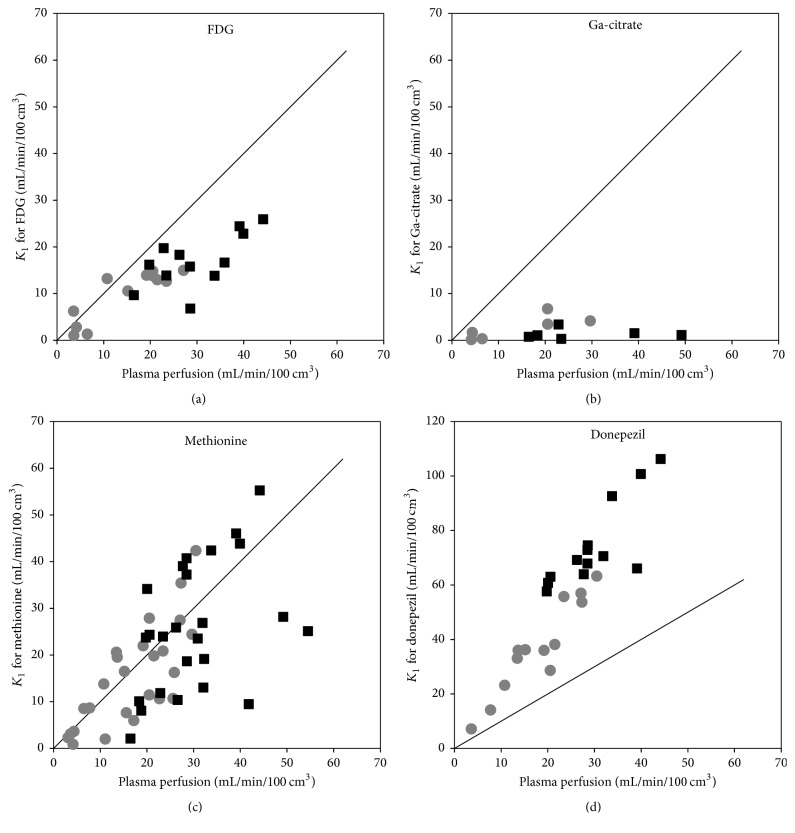
First-pass uptake *K*_1_ as a function of plasma perfusion, shown along with the identity line (*y* = *x*). The black squares represent infection foci and the grey circles represent the corresponding noninfected positions. The plots include both bone and soft tissue data. The data for [^68^Ga]Ga-citrate did not correlate with perfusion (*R*^2^ = 0.03). For [^11^C]donepezil, all data points are above the identity line, paradoxically corresponding to an extraction fraction above 100%. See Discussion.

Figure 10 shows the values of *K*_1_ plotted as a function of plasma perfusion. Notably, [^68^Ga]Ga-citrate shows only small uptake compared to perfusion, that is, a small extraction fraction.

The paradoxical plot for [^11^C]donepezil, showing *K*_1_ values that are significantly higher than perfusion (corresponding to >100% extraction), is not a result of using the simple 1TCM rather than one of the 2TCM models. Overall, the *K*_1_ data from these models (not shown) were very similar, in some cases even higher, resulting in very similar plots (not shown). For further explanations, see Discussion.

For [^18^F]FDG, [^11^C]methionine, and [^11^C]donepezil, a paired* t*-test showed a higher *K*_1_ in the infected (right) side than in the noninfected (left) side (*p* ≤ 0.0001). For [^68^Ga]Ga-citrate, no significant difference was found (*p* > 0.05).

### 3.5. Irreversible Net Uptake Rate (*K*_*i*_) for [^18^F]FDG and [^68^Ga]Ga-Citrate

For both [^18^F]FDG and [^68^Ga]Ga-citrate, good agreement was observed between *K*_*i*_ calculated from the Patlak plots and from the irr2TCM model (data not shown).

**Figure 11:**
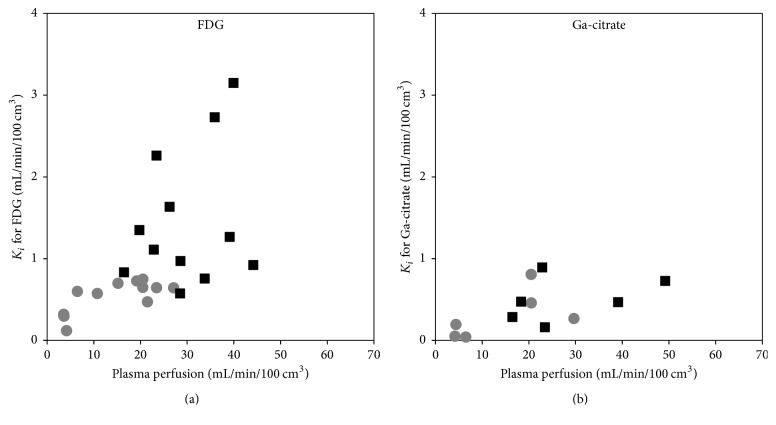
Irreversible uptake *K*_*i*_ as a function of plasma perfusion, for the tracers showing irreversible uptake: [^18^F]FDG (a) and [^68^Ga]Ga-citrate (b). Black squares represent infection foci and grey circles corresponding to noninfected positions (compare with Figure 10).

**Figure 12:**
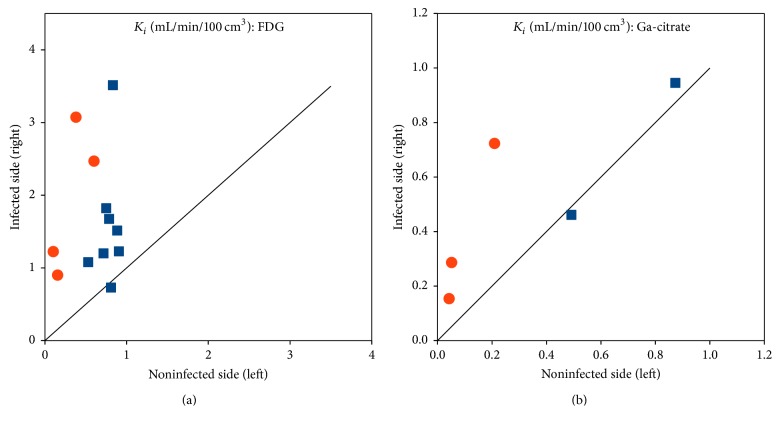
Comparison of irreversible net uptake rate *K*_*i*_ in infected (right hind limb) versus noninfected (left hind limb) positions. Bone is represented by blue squares and soft tissue by red circles. The line is the identity line (*y* = *x*). (a) shows [^18^F]FDG and (b) shows [^68^Ga]Ga-citrate; note the difference in scales.

Figure 11 shows *K*_*i*_ as a function of plasma perfusion.  Figure 12 compares *K*_*i*_ in the infected versus corresponding noninfected positions. For [^18^F]FDG, *K*_*i*_ values were significantly higher in the infected lesions than in the corresponding noninfected positions (*p* < 0.002, paired* t*-test). For [^68^Ga]Ga-citrate, the difference was not statistically significant (*p* > 0.05).

### 3.6. Distribution Volume (DV) of Reversible Tracers

For [^11^C]donepezil fitted with the 1TCM, good agreement was observed between DV calculated from the Logan plots and from (3). For [^11^C]methionine fitted with the rev2TCM, the agreement was not as good between DV from the Logan plots and from (4); however, the lack of agreement was due to the rev2TCM giving unrealistically high values in some cases (e.g., >100 mL/cm^3^), corresponding to cases with high values of *k*_3_/*k*_4_. Excluding these cases, good agreement was observed in the DV calculations.

**Figure 13:**
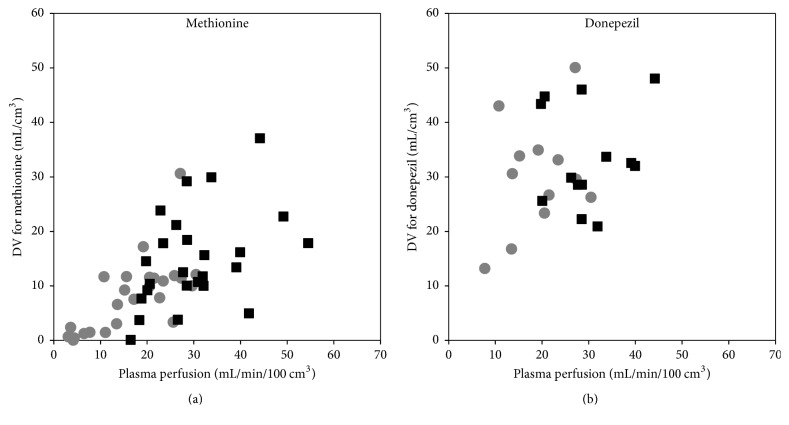
Distribution volume DV as a function of plasma perfusion, for the tracers showing reversible uptake: [^11^C]methionine (a) and [^11^C]donepezil (b). Legend as in Figure 11.

**Figure 14:**
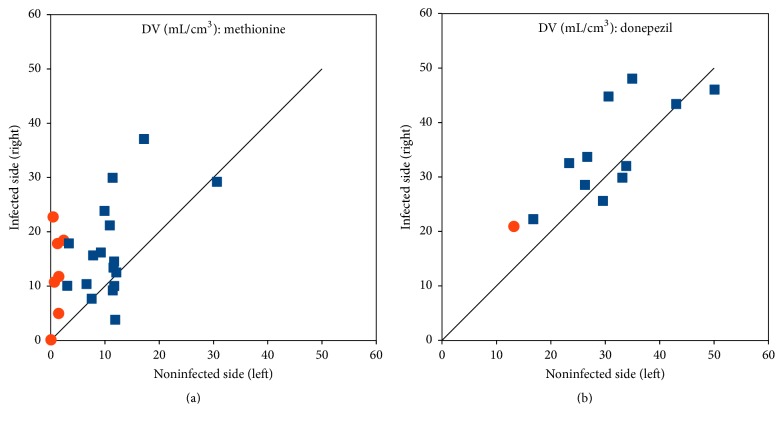
Comparison of the distribution volume DV in infected (right) and noninfected (left) limbs. Bone is represented by blue squares and soft tissue by red circles. The line is the identity line (*y* = *x*). (a) shows [^11^C]methionine, and (b) shows [^11^C]donepezil.

Overall, the slope of the Logan plot was used as a robust measure of DV.  Figure 13 shows DV as a function of plasma perfusion.  Figure 14 compares DV in the infected and corresponding noninfected locations. For [^11^C]methionine, DV was significantly higher in the infected locations (*p* = 0.0005), while the minor difference seen for [^11^C]donepezil was not significant (*p* > 0.05).

## 4. Discussion

Four very different PET tracers were studied for their potential as markers of infection in a porcine model of osteomyelitis (including associated soft tissue lesions). As noted, the results from static imaging have been reported earlier [3–5], but dynamic imaging can provide more information on the uptake processes, which is not available when the tracer concentration is measured at only one time interval.

The modelling of PET data is restricted by the length of the acquisition, the number of data points, and statistical noise. Therefore, some level of pragmatism is needed when setting up or choosing a model. Strictly irreversible uptake (in the sense of the molecules staying in the body for life) is rare, but the efflux level from a compartment may be practically zero relative to the length of the PET acquisition. In the present study, we attempted to find a level that makes the models useful for providing information about the uptake process while accepting a level of pragmatism to distinguish “practically irreversible” from “practically reversible” uptake.

### 4.1. [^18^F]FDG

For the kinetic modelling of [^18^F]FDG, the two classical models are the irr2TCM by Sokoloff et al. [36] and the rev2TCM by Phelps et al. [37]. In both models, the second tissue compartment represents the metabolite product [^18^F]FDG-6-phosphate, and in the latter model *k*_4_ > 0 represents dephosphorylation back to [^18^F]FDG. Relative to infection, the kinetic modelling of [^18^F]FDG uptake appears only to have been performed in lung studies (reviewed in [38]) and in a single study of an acute viral infection [39]. All of these studies focus on irreversible uptake models, generally the irr2TCM, although some lung studies included a separate reversible compartment for uptake in pulmonary oedema [38, 40].

In our porcine osteomyelitis model, the uptake of [^18^F]FDG was found to be (practically) irreversible, with a reasonable fit by the irr2TCM. Accordingly, the level of uptake was evaluated based on the irreversible net uptake rate, *K*_*i*_, in almost all cases showing elevated uptake in lesions compared to healthy tissue, for both OM and ST lesions (Figure 12). The correlation with perfusion seen for both first-pass uptake (Figure 10) and net uptake (Figure 11) indicates uptake of [^18^F]FDG to be more flow-limited than diffusion-limited.

### 4.2. [^68^Ga]Ga-Citrate

Dynamic PET studies of [^68^Ga]Ga-citrate and [^68^Ga]Ga-transferrin have been published before [8, 41, 42], but the present study appears to be the first to include kinetic modelling. As noted by Kumar and Boddeti [43], no literature is available on the early imaging times of [^67^Ga]Ga-citrate SPECT. We found the uptake of ^68^Ga to be well described by the irr2TCM (Figure 7). However, first-pass uptake (*K*_1_) of ^68^Ga was small compared to that of the other tracers, indicating a very small extraction fraction with little dependence on perfusion (Figures 10 and 11), that is, diffusion-limited.

The physiological reason for the slow uptake of ^68^Ga may be related to the binding of gallium to a large protein (transferrin). Uptake will either require extravasation of the large [^68^Ga]Ga-transferrin complex or require a two-step process, such as the release of  ^68^Ga from the protein before the uptake or the uptake of [^68^Ga]Ga-transferrin by leukocytes which then enter tissue [44]. Therefore, even though bacteria may show increased uptake of gallium due to its chemical similarities with iron [43], the overall uptake mechanism appears to be quite slow.

For static imaging, slow uptake favours late imaging. Physically, the half-life of ^68^Ga restricts imaging to a few hours after injection. In a study of lung lesions, Vorster et al. [10] recommended imaging to start no later than 120 minutes p.i.

Compared to other ^68^Ga infection studies [8–10, 41], the results in the porcine osteomyelitis model (present paper and [3, 4]) appear disappointing. At the basic level, the tracer is a Ga^3+^ ion (with chemical similarities to the Fe^3+^ ion), which makes a species difference unlikely. A different reason may be indicated by Figure 12: maybe the tracer is able to differentiate infected* soft tissue* from healthy tissue (despite the slow uptake rate) but is not suitable for bone infections. Mäkinen et al. [8] did find uptake in bone lesions in a rat model, but noted as a limitation of the study that their model “perhaps best simulates osteomyelitis arising from grossly contaminated long-bone fractures.” In contrast, the porcine osteomyelitis model represents haematogenous osteomyelitis without bone trauma.

### 4.3. [^11^C]Methionine

The modelling of [^11^C]methionine uptake required a rev2TCM. For comparison, Fischman et al. [45] described the muscle uptake of [^11^C]methionine with a 2TCM, where the second compartment represented the incorporation of [^11^C]methionine in tissue proteins, and they assumed that the degradation rate of labelled protein could be ignored (corresponding to *k*_4_ = 0 in our notation). That is, they suggested an irr2TCM rather than rev2TCM. Our finding of rev2TCM as preferable thus corresponds to protein degradation being nonnegligible.

Despite the considerable metabolism of [^11^C]methionine during the acquisition time (Figure 3), practically only the *k*_4_ rate constant depended on whether modelling was based on the uncorrected or the metabolite-corrected input function (with higher *k*_4_ values in the latter case). It appears that the metabolite products have kinetics quite similar to the original molecule. The correlation with perfusion seen for both first-pass uptake (Figure 10) and distribution volume (Figure 13) indicates uptake of [^11^C]methionine to be flow-limited.

The unexpected rise in the plasma activity curves after ~20 minutes (Figure 5) might be explained by a heavy uptake by metabolizing organs (the liver), followed by a later release of radioactive metabolite products to the blood pool.

Generally, the distribution volume (DV) for [^11^C]methionine was higher in the infected tissue than in the noninfected tissue (Figure 14). This difference between infected and noninfected tissue could point to [^11^C]methionine having a role in infection imaging (regardless of the role of perfusion in causing the distinction). In line with the results from static imaging [3, 5], the distinction between infected and noninfected tissue appeared more clear for soft tissue than for bone (Figure 14). As indicated by the two already mentioned case reports [12, 13], [^11^C]methionine could be useful for brain infection imaging, where [^18^F]FDG suffers from the high physiological uptake in healthy brain tissue.

The uptake curves in both infected and noninfected tissues appear quite stable after approximately 10–15 minutes (Figure 8), indicating that static imaging could be performed starting at this time.

### 4.4. [^11^C]Donepezil

Metabolite-corrected [^11^C]donepezil could be modelled with the 1TCM model. However, the *K*_1_ parameter was systematically higher than plasma (and blood) perfusion, paradoxically indicating an extraction fraction above 100% (Figure 10). We consider this to be an indication that at least one of the radioactive metabolite products of [^11^C]donepezil has marked uptake along with the main tracer. Indeed, Funaki et al. [46] reported that affinity of the M1 metabolite for AChE is almost as high as the affinity of donepezil for AChE; the M1 metabolite is radioactive when the parent tracer is [5-^11^C-*methoxy*]donepezil. In a steady-state study with a regular administration of donepezil, Meier-Davis et al. [47] found M1 to be relatively more prominent in minipigs than in humans and rats.

Meier-Davis et al. [47] also found the overall level of metabolites to be higher in the pigs. This species difference may explain why we found relatively fast metabolization of [^11^C]donepezil (Figure 4), in contrast to the human study by Hiraoka et al. [48] who saw only minor metabolism of [^11^C]donepezil (>85% remaining after 30 minutes) and therefore did not need metabolite correction. Also, Hiraoka et al. found the rev2TCM to be unequivocally better than the 1TCM, while our study finds the distinction less clear. As noted, however, their input functions were not corrected for metabolites.

The strong correlation between first-pass uptake and perfusion (Figure 10) indicates flow-limited uptake of [^11^C]donepezil, although the effect is less evident for distribution volume (Figure 13).

Regarding [^11^C]donepezil as an infection tracer, our data did show an overall higher first-pass uptake in the infected lesions and a strong correlation with perfusion (Figure 10), while the DV was only slightly higher in the infected sites than in the corresponding noninfected locations (Figure 14), and the difference was not statistically significant.

### 4.5. Limitations

The pig model was developed as a model for osteomyelitis, for which reason only relatively few soft tissue lesions were available, limiting the scope of the study as a general infection study. For [^68^Ga]Ga-citrate, only relatively limited data were available.

## 5. Conclusion

[^18^F]FDG was reasonably well described by the irr2TCM (irreversible uptake, three rate constants) for the 60-minute length studied, and, for both bone and soft tissue, [^18^F]FDG showed increased uptake in infected tissue (Figure 12). The correlation with perfusion indicated that the tracer is mainly flow-limited.

[^68^Ga]Ga-citrate was also well described by irrTCM but showed very little or very slow uptake, which was a limitation for infection imaging. The difference between infected and noninfected sites appeared to be higher in soft tissue rather than bone lesions, but too little data were available to draw a conclusion (Figure 12). Uptake was slow and diffusion-limited. To allow time for uptake, “late” imaging is preferable, but not so late that the radionuclide has decayed. Imaging at 120 minutes p.i. appears a good compromise.

[^11^C]methionine needed a rev2TCM (reversible uptake, four rate constants) for modelling. Despite considerable metabolism during the 40 minutes modelled, the *K*_1_, *k*_2_, and *k*_3_ rate constants were only slightly affected if an uncorrected input function was used, that is, only *k*_4_ was markedly affected. In a majority of cases, the uptake (measured as the distribution volume) was elevated in the infected tissue compared to the noninfected tissue (Figure 14), but the difference was less than for [^18^F]FDG (measured as the net uptake rate, Figure 12). Uptake appeared flow-limited. Based on the activity curves, imaging at ~15 minutes p.i. appears favourable.

[^11^C]donepezil could be modelled with a 1TCM (reversible uptake, two rate constants) but required metabolite correction—at least in this juvenile, porcine model. The uptake of labelled metabolite products appeared to be nonnegligible. Based on the present study, the uptake of [^11^C]donepezil in osteomyelitis seems to depend more on perfusion (flow-limited) than on differences between infected and noninfected tissues. For soft tissue infection, too few data were available to draw a conclusion.

Overall, among the studied PET tracers [^18^F]FDG showed optimal characteristics for the detection of infectious foci. [^68^Ga]Ga-citrate and [^11^C]donepezil were not found to be useful for imaging of osteomyelitis. For soft tissue, [^11^C]methionine and perhaps [^68^Ga]Ga-citrate may be applicable to quantify different aspects of inflammatory or infectious processes, while too few soft tissue data on [^11^C]donepezil were available to draw any conclusions.

## Figures and Tables

**Table 1:** Pig ID numbers (1–11) for different tracers and lesions^a^.

Tracer	Dynamic data available	Osteomyelitic (OM) lesions^b^				Soft tissue (ST) lesions^b^
		Proximal femur	Distal femur	Proximal tibia	Distal tibia	
[^15^O]water [6]	1–11	1	1, 5–10	1, 6–10	7, 8, 10	1, 2, 4^c^, 8–11
[^18^F]FDG	1–7, 9, 11	1	5–7	6, 7, 9	7	1, 4, 9, 11
[^68^Ga]Ga-citrate	1–5	1	5	—	—	1, 2, 4
[^11^C]methionine	1–10	1	1, 5–10	1, 6–10	7, 8, 10	1, 2, 4^c^, 8–10
[^11^C]donepezil	5–7, 9, 10	—	5–7, 9, 10	6, 7, 9, 10	7, 10	9, 10

^a^Numbering as presented in the [^15^O]water paper [6]. ^b^The[^15^O]water paper presents an exhaustive list of the lesions studied. In pig number 3, inoculation failed to produce infection [3]. ^c^Two soft tissue lesions within the FOV (pig number 4).

**Table 2:** Evaluation of the fitting models for the investigated tracers and input functions.

Tracer	Number of OM + ST foci modelled	Plasma input function	Visual impression of fit	Linear Patlak plot	Lowest AIC_c_
[^18^F]FDG	8 + 7	Uncorrected	Good fit of both 2TCM, with rev2TCM giving only minor improvement over irr2TCM. Poor fit of 1TCM.	Yes	rev2TCM

[^68^Ga]Ga-citrate	2 + 3	Uncorrected	Good fit of both 2TCM, with 1TCM reasonable for some curves.	Yes	irr2TCM or rev2TCM

[^11^C]methionine	17 + 7	Uncorrected	Good fits of rev2TCM. Cases with good irr2TCM fit (generally low-lying curves).	No	See main text
		Metab. corr.	For irr2TCM, the fit was improved over the uncorrected input function. Overall, rev2TCM appeared best.	Almost	

[^11^C]donepezil	11 + 2	Uncorrected	Poor fits for all models.	No	See main text
		Metab. corr.	Good fits with 1TCM, only slightly improved by 2TCM (irr or rev).	Generally not	

## References

[1] Basu S., Chryssikos T., Moghadam-Kia S., Zhuang H., Torigian D. A., Alavi A. (2009). Positron emission tomography as a diagnostic tool in infection: present role and future possibilities. *Seminars in Nuclear Medicine*.

[2] Ahmad Sarji S. (2006). Physiological uptake in FDG PET simulating disease. *Biomedical Imaging and Intervention Journal*.

[3] Nielsen OL., Afzelius P., Bender D. (2015). Comparison of autologous ^111^In-leukocytes, ^18^F-FDG, ^11^C-methionine, ^11^C-PK11195 and ^68^Ga-citrate for diagnostic nuclear imaging in a juvenile porcine haematogenous staphylococcus aureus osteomyelitis model. *American Journal of Nuclear Medicine and Molecular Imaging*.

[4] Afzelius P., Nielsen O. L., Alstrup A. K. O. (2016). Biodistribution of the radionuclides ^18^F-FDG, ^11^C-methionine, ^11^C-PK11195, and ^68^Ga-citrate in domestic juvenile female pigs and morphological and molecular imaging of the tracers in hematogenously disseminated *Staphylococcus aureus* lesions. *American Journal of Nuclear Medicine and Molecular Imaging*.

[5] Afzelius P., Alstrup A. K. O., Schønheyder H. C. (2016). Utility of ^11^C-methionine and ^11^C-donepezil for imaging of *Staphylococcus aureus* induced osteomyelitis in a juvenile porcine model: comparison to autologous ^111^In-labelled leukocytes, ^99m^Tc-DPD, and ^18^F-FDG. *American Journal of Nuclear Medicine and Molecular Imaging*.

[6] Jødal L., Nielsen O. L., Afzelius P., Alstrup A. K. O., Hansen S. B. (2017). Blood perfusion in osteomyelitis studied with ^[15^O]water PET in a juvenile porcine model. *EJNMMI Research*.

[7] Jensen S. B., Nielsen K. M., Mewis D., Kaufmann J. (2013). Fast and simple one-step preparation of ^68^Ga citrate for routine clinical PET. *Nuclear Medicine Communications*.

[8] Mäkinen T. J., Lankinen P., Pöyhönen T., Jalava J., Aro H. T., Roivainen A. (2005). Comparison of ^18^F-FDG and ^68^Ga PET imaging in the assessment of experimental osteomyelitis due to *Staphylococcus aureus*. *European Journal of Nuclear Medicine and Molecular Imaging*.

[9] Nanni C., Errani C., Boriani L. (2010). ^68^Ga-citrate PET/CT for evaluating patients with infections of the bone: preliminary results. *Journal of Nuclear Medicine*.

[10] Vorster M., Maes A., Jacobs A. (2014). Evaluating the possible role of ^68^Ga-citrate PET/CT in the characterization of indeterminate lung lesions. *Annals of Nuclear Medicine*.

[11] Zhao S., Kuge Y., Kohanawa M. (2008). Usefulness of ^11^C-methionine for differentiating tumors from granulomas in experimental rat models: a comparison with ^18^F-FDG and ^18^F-FLT. *Journal of Nuclear Medicine*.

[12] Hirata K., Shiga T., Fujima N. (2012). ^11^C-methionine positron emission tomography may monitor the activity of encephalitis. *Acta Radiologica*.

[13] Maeda Y., Oguni H., Saitou Y. (2003). Rasmussen syndrome: multifocal spread of inflammation suggested from MRI and PET findings. *Epilepsia*.

[14] Morooka M., Kubota K., Kadowaki H. (2009). ^11^C-methionine PET of acute myocardial infarction. *Journal of Nuclear Medicine*.

[15] Sugimoto H., Ogura H., Arai Y., Iimura Y., Yamanishi Y. (2002). Research and development of donepezil hydrochloride, a new type of acetylcholinesterase inhibitor. *Japanese Journal of Pharmacology*.

[16] Kawashima K., Fujii T., Moriwaki Y., Misawa H. (2012). Critical roles of acetylcholine and the muscarinic and nicotinic acetylcholine receptors in the regulation of immune function. *Life Sciences*.

[17] Fujii T., Watanabe Y., Fujimoto K., Kawashima K. (2003). Expression of acetylcholine in lymphocytes and modulation of an independent lymphocytic cholinergic activity by immunological stimulation. *Biogenic Amines*.

[18] Jørgensen N. P., Alstrup A. K. O., Mortensen F. V. (2017). Cholinergic PET imaging in infections and inflammation using ^11^C-donepezil and ^18^F-FEOBV. *European Journal of Nuclear Medicine and Molecular Imaging*.

[19] Johansen L. K., Koch J., Frees D. (2012). Pathology and Biofilm Formation in a Porcine Model of Staphylococcal Osteomyelitis. *Journal of Comparative Pathology*.

[20] Johansen L. K., Svalastoga E. L., Frees D. (2013). A new technique for modeling of hematogenous osteomyelitis in pigs: Inoculation into femoral artery. *Journal of Investigative Surgery*.

[21] Alstrup A. K. O., Nielsen K. M., Schønheyder H. C. (2016). Refinement of a hematogenous localized osteomyelitis model in pigs. *Scandinavian Journal of Laboratory Animal Science*.

[22] Jødal L., Hansen S. B., Jensen S. B. (2016). Impact of contamination with long-lived radionuclides on PET kinetics modelling in multitracer studies. *Nuclear Medicine Communications*.

[23] Som P., Atkins H. L., Bandoypadhyay D. (1980). A fluorinated glucose analog, 2-fluoro-2-deoxy-D-glucose (F-18): Nontoxic tracer for rapid tumor detection. *Journal of Nuclear Medicine*.

[24] Tsan M.-F. (1985). Mechanism of gallium-67 accumulation in inflammatory lesions. *Journal of Nuclear Medicine*.

[25] Van den Hoff J., Burchert W., Muller-Schauenburg W., Meyer G.-J., Hundeshagen H. (1993). Accurate local blood flow measurements with dynamic PET: Fast determination of input function delay and dispersion by multilinear minimization. *Journal of Nuclear Medicine*.

[26] Patlak C. S., Blasberg R. G., Fenstermacher J. D. (1983). Graphical evaluation of blood-to-brain transfer constants from multiple-time uptake data. *Journal of Cerebral Blood Flow and Metabolism*.

[27] Patlak C. S., Blasberg R. G. (2016). Graphical Evaluation of Blood-to-Brain Transfer Constants from Multiple-Time Uptake Data. Generalizations. *Journal of Cerebral Blood Flow & Metabolism*.

[28] Innis R. B., Cunningham V. J., Delforge J. (2007). Consensus nomenclature for *in vivo* imaging of reversibly binding radioligands. *Journal of Cerebral Blood Flow & Metabolism*.

[29] Logan J., Fowler J. S., Volkow N. D. (1990). Graphical analysis of reversible radioligand binding from time-activity measurements applied to [N-^11^C-methyl]-(-)-cocaine PET studies in human subjects. *Journal of Cerebral Blood Flow & Metabolism*.

[30] Logan J. (2000). Graphical analysis of PET data applied to reversible and irreversible tracers. *Nuclear Medicine and Biology*.

[31] Thiele F., Buchert R. (2008). Evaluation of non-uniform weighting in non-linear regression for pharmacokinetic neuroreceptor modelling. *Nuclear Medicine Communications*.

[32] Yaqub M., Boellaard R., Kropholler M. A., Lammertsma A. A. (2006). Optimization algorithms and weighting factors for analysis of dynamic PET studies. *Physics in Medicine and Biology*.

[33] TPC. List of applications in TPCCLIB. *Turku PET Centre web site*. Available from: http://www.turkupetcentre.net/programs/doc/index.html

[34] Akaike H. (1974). A new look at the statistical model identification. *IEEE Transactions on Automatic Control*.

[35] Burnham K. P., Anderson D. R. (2004). Multimodel inference: understanding AIC and BIC in model selection. *Sociological Methods and Research*.

[36] Sokoloff L., Reivich M., Kennedy C. (1977). The [^14^C]deoxyglucose method for the measurement of local cerebral glucose utilization: theory, procedure, and normal values in the conscious and anesthetized albino rat. *Journal of Neurochemistry*.

[37] Phelps M. E., Huang S. C., Hoffman E. J., Selin C., Sokoloff L., Kuhl D. E. (1979). Tomographic measurement of local cerebral glucose metabolic rate in humans with (F-18)2-fluoro-2-deoxy-D-glucose: validation of method. *Annals of Neurology*.

[38] Schroeder T., Vidal Melo M. F., Venegas J. G. (2011). Analysis of 2-[Fluorine-18]-Fluoro-2-deoxy-D-glucose uptake kinetics in PET studies of pulmonary inflammation. *Academic Radiology*.

[39] Chefer S., Thomasson D., Seidel J. (2015). Modeling [^18^F]-FDG lymphoid tissue kinetics to characterize nonhuman primate immune response to Middle East respiratory syndrome-coronavirus aerosol challenge. *EJNMMI Research*.

[40] Schroeder T., Vidal Melo M. F., Musch G., Harris R. S., Venegas J. G., Winkler T. (2008). Modeling Pulmonary Kinetics of 2-Deoxy-2-[^18^F]fluoro-d-glucose During Acute Lung Injury. *Academic Radiology*.

[41] Kumar V., Boddeti D. K., Evans S. G., Roesch F., Howman-Giles R. (2011). Potential use of ^68^Ga-apo-transferrin as a PET imaging agent for detecting Staphylococcus aureus infection. *Nuclear Medicine and Biology*.

[42] Thackeray J. T., Bankstahl J. P., Wang Y. (2014). Targeting post-infarct inflammation by PET imaging: comparison of ^68^Ga-citrate and ^68^Ga-DOTATATE with ^18^F-FDG in a mouse model. *European Journal of Nuclear Medicine and Molecular Imaging*.

[43] Kumar V., Boddeti D. K., Baum R. P., Rösch F. (2013). ^68^Ga-radiopharmaceuticals for PET imaging of infection and inflammation. *Theranostics, Gallium-68, and Other Radionuclides*.

[44] Pauwels E. K. J., McCready V. R., Stoot J. H. M. B., Van Deurzen D. F. P. (1998). The mechanism of accumulation of tumour-localising radiopharmaceuticals. *European Journal of Nuclear Medicine*.

[45] Fischman A. J., Yu Y. M., Livni E. (1998). Muscle protein synthesis by positron-emission tomography with L-[methyl- ^11^C]methionine in adult humans. *Proceedings of the National Academy of Sciences of the United States of America*.

[46] Funaki Y., Kato M., Iwata R. (2003). Evaluation of the binding characteristics of [5-^11^C-methoxyl]donepezil in the rat brain for in vivo visualization of acetylcholinesterase. *Journal Pharmacological Sciences*.

[47] Davis S. R., Murgasova R. (2012). Comparison of metabolism of donepezil in rat, mini-pig and human, following oral and transdermal administration, and in an in vitro model of human epidermis. *Journal of Drug Metabolism & Toxicology*.

[48] Hiraoka K., Okamura N., Funaki Y. (2009). Quantitative analysis of donepezil binding to acetylcholinesterase using positron emission tomography and [5-^11^C-methoxy]donepezil. *NeuroImage*.

